# Megakaryocytes in Bone Metastasis: Protection or Progression?

**DOI:** 10.3390/cells8020134

**Published:** 2019-02-08

**Authors:** Paola Maroni

**Affiliations:** IRCCS Istituto Ortopedico Galeazzi, 20161 Milan, Italy; paola.maroni@grupposandonato.it; Tel.: +39-02-6621-4759

**Keywords:** megakaryocytes, bone metastasis, bone marrow, microenvironment, platelets

## Abstract

Bone is the primary site where some cancers develop secondary growth, particularly those derived from breast and prostate tissue. The spread of metastasis to distant sites relies on complex mechanisms by which only cells endowed with certain characteristics are able to reach secondary growth sites. Platelets play a pivotal role in tumour growth, by conferring resistance to shear stress to the circulating tumour cells and protection against natural killer cell attack. Mature polyploid megakaryocytes (MKs) reside in close proximity to the vascular sinusoids of bone marrow, where their primary function is to produce platelets. Emerging evidence has demonstrated that MKs are essential for skeletal homeostasis, due to the expression and production of the bone-related proteins osteocalcin, osteonectin, bone morphogenetic protein, osteopontin, bone sialoprotein, and osteoprotegerin. Debate surrounds the role that MKs play in the development of bone metastasis, which is the topic of this mini-review.

## 1. Introduction

Megakaryocytes (MKs), polyploid cells derived from bone marrow-residing hematopoietic stem cells (HSC) in response to thrombopoietin (TPO), are responsible for the production of platelets through a complicated regulatory mechanism. A complex network of cellular interactions and the typesetting of the extracellular matrix (ECM) may be responsible for the differentiation and maturation of MKs in bone marrow, where the microenvironment is a key factor in megakaryopoiesis.

MKs are also involved in the regulation of bone homeostasis: they influence proliferation and differentiation of osteoclasts (OCs) and osteoblasts (OBs) and secrete the bone matrix proteins osteocalcin, osteonectin, bone sialoprotein, and osteopontin [1,2,3,4,5].

As MKs have a central role in the regulation of bone mass and a strategic position in bone marrow, they have come into the focus of recent research on their possible involvement in the development of bone metastasis.

Bone marrow is a common site for metastasis formation, because it provides a receptive microenvironment for malignant cells, where they can find supportive stromal cells, growth factors, and metabolic components, as well as the blood vessels that supply nutrients and oxygen and provide for waste disposal.

The question of whether MKs play a pro- or anti-metastatic role in bone marrow is widely debated. Many studies report a protective role of MKs against bone metastasis development [6,7,8], while others suggest that MKs promote bone metastasis [9,10].

The aim of this review is to provide a brief overview of the involvement of MKs in bone metastasis, and to integrate the data from the literature with my observations from studies in which I have focused on the role of MKs in bone marrow and in the bone microenvironment following the engraftment of cancer cells.

## 2. Megakaryocytes: Relationship with the Bone Marrow Extracellular Matrix

The bone marrow ECM exhibits peculiar characteristics, owing to its meshwork of secreted proteins that provide structural support (fibrillar and non-fibrillar collagen, laminin, fibronectin (FN), and fibrinogen) and biochemical cues for cells in the bone marrow.

As MKs interact in with the various ECM proteins within the bone marrow, this seems to orchestrate their maturation in specific sites. Moreover, the presence of specific ECM components (mainly collagen and FN) around mature MKs in bone marrow has prompted exploration into the possibility that MKs themselves release ECM proteins. Indeed, human MKs possess collagen synthesis mechanisms, and produce and release FN as well as type III and IV collagen [11]. The adhesion of MKs to type I collagen promotes in vitro MK expansion and spread, but this interaction inhibits pro-platelet formation through the activation of integrin α2β1 and the Rho-ROCK axis. In contrast, type III and IV collagen stimulate pro-platelet production through the PI3K/Akt signalling pathway [12].

FN is essential for the proliferation, differentiation, and maintenance of megakaryocytic lineage cells. When MKs interact with FN through VLA-4 and VLA-5 (FN receptors), pro-platelet formation is increased [13]. Cellular FN (cFN) produced by human MKs promotes their anchoring to type I collagen. This process suggests that cFN modulates the signalling (biochemical and mechanical) underlying the interaction between MKs and type I collagen [14].

Glycosaminoglycans (GAGs), which are abundant in the bone marrow ECM, are critical for the development of MKs. Hyaluronic acid appears to be an inhibitor of platelet production by MKs, while dermatan sulphate increases pro-platelet production in MKs stimulated with TPO [15,16].

Recent studies have shown that the stiffness of the bone marrow ECM is vital for MK maturation and pro-platelet formation [17]. MKs are able to detect the physical properties of the ECM through mechanosensitive ion channels: a rigid ECM is adverse to MK maturation and pro-platelet release [17].

The relationship between MKs and the microenvironment is bidirectional. MKs are able to secrete a host of other bone matrix proteins, including osteocalcin, osteonectin/SPARC, bone sialoprotein, and osteopontin [1,2,3,4,5]. These matricellular proteins are associated with the ECM, and serve more to fine-tune the cell–ECM interactions and cellular functions rather than exert a structural function. For example, SPARC regulates processes involved in the assembly of the structural proteins of the ECM, in addition to conditioning osteoblast (OB) and osteoclast (OC) differentiation. Furthermore, the ECM microenvironment is involved in regulating the release of pro-platelets into the bone marrow vasculature. Malara and colleagues demonstrated that, among bone marrow ECM components, FN, type IV collagen, and laminin are the most abundant in the sinusoid area of the bone marrow and make up the pericellular matrix surrounding the MKs. For the first time, they demonstrated that mouse MKs express basement membrane components both in vitro and in vivo. This function is important for the regulation of basement membrane activity in physiological and pathological conditions, as well as for bone marrow ECM homeostasis [11].

Of note, the matricellular proteins of ECM are essential in the modulation of multiple aspects of tumour biology (growth, progression) and in secondary growth of tumours into the bone [18]. The matricellular proteins not only support malignant cells, but also remodel the bone microenvironment and favour tumour progression.

## 3. Megakaryocyte Maturation and Platelet Production

**Figure 1:**
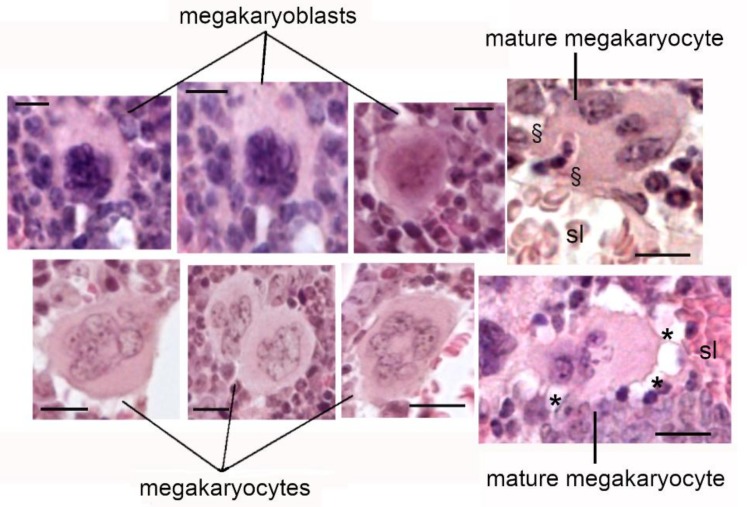
Megakaryocyte (MK) development is a multiphase process divided in four stages: megakaryoblasts, stages I and II; MKs, stages III and IV. sl: sinusoid lumen; *: cytoplasmic processes releasing pro-platelets into the blood stream; **§**: large protrusion entering the sinusoid lumen. Scale bar = 10 μm.

The development of MKs is a multiphase process that takes place through four stages, each of which characterized by morphologic changes. In stage I, the proliferating MKs are round and contain a regular nucleus and scarce cytoplasm. In stage II, the immature MKs have an irregular shape and small pseudopodia, and synthesize the huge amounts of proteins needed to generate MK-characteristic structures. In stages III and IV, MKs become non-proliferating cells endowed with an irregular shape and a large nucleus. MKs can also be multinucleated with pyknotic nuclei and prominent granular cytoplasm (Figure 1). In the last stage, terminally differentiated MKs extrude long cytoplasmic processes into the sinusoid lumen through gaps in the endothelium, and release pro-platelets into the blood stream [19]. Mature MKs are, in fact, present in a specialized area of the bone marrow in close proximity to the vascular sinusoid, where they are subjected to changes that lead to the release of platelets.

In a recent and elegant paper, Brown et al. develop an approach to visualize MKs in bone marrow using intravital correlative light electron microscopy (CLEM) combined with ultrastructural details obtained with large-volume electron tomography. They demonstrated a novel mechanism for the intravasation of MK materials into the marrow sinusoids as a large protrusion (from 4 to 10 μm) instead of as pro-platelets (≤3 μm). These large protrusions are not usually formed from MKs in vitro, and the authors suggest that some elements of the bone marrow microenvironment are crucial for their generation [20].

TPO is the primary regulator of megakaryocytopoiesis and platelet production; it is essential for the full maturation of MKs [21]. TPO is normally synthesized and secreted by hepatocytes, but can also be produced by the OBs in the bone marrow [22].

Shear stress remodels mature MKs to produce MK particles: platelet-like particles (PLPs), pro-platelets, and MK microplatelets (MKMPs) [23]). Jiang and colleagues report that on entering the bone marrow sinusoids, the MKs are exposed to hemodynamic stresses, an event that enhances platelet generation and production of MKMP. The authors demonstrate that MKMPs promote MK survival and the differentiation of HSCs versus MK lineage also in the absence of TPO, probably through the delivery of RNAs [24]. These two studies [23,24], however, were performed only in reconstructed in vitro experiments, and their physiological relevance is debatable.

Stegner et al., in fact, utilizing recently developed imaging techniques (in vivo and in vitro), analysed the distribution of MKs in the bone marrow and demonstrated that MKs do not enter the bone marrow sinusoid; rather, they remain in the bone marrow, and only the pro-platelets are released into the circulation and are exposed to shear forces from blood flow [25]. In agreement, other authors reported that MKs predominantly enter sinusoids as a large protrusion [20].

Xiao et al. demonstrated an essential role of OBs in the bone marrow microenvironment and megakaryopoiesis [26]. OBs support CD41+ Sca-1+ MK expansion, megakaryopoiesis, and platelet formation through IL-9 production, which triggers IL-9 receptor (IL-9R)/Stat3 signalling.

GATA-1, FOG-1, and Nf-E2 are key transcription factors for the regulation of the stages in MK differentiation; specifically, GATA-1 and Nf-E2 are required for the terminal differentiation of MKs and for platelet production [27]. Mice deficient in the transcription factors GATA-1 or Nf-E2 exhibit a significant increase in the number of immature MKs, with a concomitant reduction in the number of platelets and a marked increase in trabecular and cortical bone mass (osteosclerosis) [28]. 

The “classical concept” of megakaryopoiesis posits the existence of two physiologically distinct HSC niches: the endosteal (or osteoblastic) niche and the vascular niche. The endosteal niche, located at the bone/bone-marrow interface, promotes stem cell quiescence, whereas the vascular niche supports the expansion of haematopoietic lineages; in particular, it is critical for MK function and platelet production.

A recent paper [25] describes a modified model for megakaryopoiesis, where MKs do not need to migrate to reach blood vessels, but where MKs at the sinusoids are replenished by a sinusoidal precursor located in close proximity to the vasculature. The authors hypothesize the existence of two pools of MKs: one resident near the vessels, readily active to produce platelets (fast responders); the other, distant from the vasculature, consisting of quiescent MKs, or MK progenitor cells (slow responders) [25]. Currently, the emerging idea is that “vascular” and “endosteal” niches are not completely separable, but rather that a random distribution of immature or mature MKs exists in small microenvironments throughout the bone marrow [25].

The release of platelets from MKs is a complex mechanism, in which the cytoplasm of MKs is converted into long processes, called pro-platelets, which are released into the bloodstream. MKs and platelets carry a peculiar cargo of α-granules, dense bodies, and lysosomes [29]. The stored soluble factors are synthesized in the intracellular compartment of the MKs, and are also endocytosed or pinocytosed from the microenvironment. Therefore, as concerns the bone marrow microenvironment, it is important to define the protein content of MKs and platelets either by altering MKs gene transcription or by making the proteins for cellular uptake available [9]. It was reported that genetic information is transferred in the form of mRNAs and miRNAs from MKs to platelets; thus, it seems that MKs also contribute to the diversification of the rich repertoire of the RNA species in circulating platelets [30]. The “genetic code” in MKs is prone to change in acute disease, genetic mutation, and by race. These changes can be transferred into developing platelets [30].

### 3.1. Role of Tumour Suppressor p53 and Megakaryopoiesis

Tumour suppressor p53 is important for regulating MK differentiation from HSC to produce mature MKs. The tumour suppressor p53 is also responsible for polyploidization and apoptosis, two integral components of MK differentiation. In addition, “p53 regulon”, which includes the cell cycle-regulator p21 and the apoptosis inducer Bax, is thought to orchestrate these effects. [31,32]. After specific post-translational modifications like acetylation, p53 selects the promoters of genes for either cell cycle arrest or apoptosis. A recent paper hypothesized that acetylation of p53 at lysine sites (K320, K373, K382) might occur in the response of MKs to shear stress [23]. Acetylated p53 binds Bax, the mitochondria release cytochrome c, and Caspase 9 is activated. The apoptosome with activated Caspase 9 can cleave and activate executioner caspase (Caspase 3). The study demonstrated for the first time that, in MKs subjected to shear flow, Caspase 3 activation is also important for the biogenesis of MK particles (pro-platelets, PLPs, and MKMPs) [23].

### 3.2. Platelets and Cancer

Platelets have recently received particular attention, since they have been recognized to be associated with the progression of cancer from primary tumours to secondary metastatic outbreaks at different steps [33]. Platelets promote the survival of circulating tumour cells during haematogenous dissemination, thus protecting them from shear stress, host immune attack (natural killer cells), and apoptosis. Moreover, activated platelets release transforming growth factor β (TGF-β), which aids in promoting the trans-differentiation of circulating tumour cells into mesenchymal-like phenotypes through the Smad signalling pathway [34]. Finally, platelets also participate later in the metastatic process by promoting the extravasation of tumour cells and the localization of circulating tumour cells in a secondary growth site [35,36]. 

Accumulating evidence supports the concept that platelets and tumour cells, through direct cellular contact, maintain reciprocal interactions within the tumour microenvironment where bidirectional cross-talk between tumour cells and platelets activates cell surface receptors, the release of soluble proteins, and the shedding of microparticles by platelets. The loop of reciprocal activation results in enhanced tumour cell attachment, proliferation, and spread [37]. Cancer cells can also “educate” platelets by modulating the RNA profiles and phenotype. The platelet RNA profile has been demonstrated to change in different types of cancer (lung, prostate, breast cancer, and glioma) [38,39,40]. Interestingly, knowing the tumour-educated platelet-mRNA profile helps gain an idea of the status of metastatic lesions, and is useful for the stratification of patients for appropriate personalized molecular therapy [41].

Given the pivotal roles of platelets in cancer progression, targeting platelet–cancer cell interaction is a potential strategy to counteract cancer metastasis and to overcome drug resistance [42]. Numerous drugs have been developed to target platelet receptors, to interfere with granule release from platelets, or to inhibit platelet-specific enzymes. The use of these drugs in clinical and in pre-clinical studies is limited principally by their interference with haemostasis, and because the interaction between cancer cells and platelets is not yet fully understood [33]. Therefore, approaches that specifically target platelet interaction with tumour cells, without interfering with normal platelet function, could provide a potential therapeutic tool [33].

## 4. Role of Megakaryocytes in Bone Homeostasis

MKs play a critical role in regulating bone mass; in vivo and in vitro evidence has shown that MKs can have a significant effect on the proliferation and differentiation of OCs and OBs, thus influencing skeletal homeostasis. The deficiency of GATA-1 or Nf-E2 in mice not only hampered MK maturation and reduction in platelet number/release, but also largely increased the number of OBs. In vitro data indicate that cell-to-cell contact between immature MKs and OB is required to stimulate OB proliferation and activation, with a consequent increase in bone mass. These observations by Kacena et al. suggest a novel function for MK–OB interactions in bone homeostasis [28].

MKs regulate bone tissue deposition by modulating both formation and resorption: MKs secrete a factor that inhibits OC formation. At the same time, MKs stimulate OB proliferation and differentiation [43]. Several factors are responsible for these functions. MKs secrete proteins that inhibit osteoclastogenesis and bone resorption (e.g., Granulocyte-macrophage colony-stimulating factor (GM-CSF), osteoprotegerin, IL-10, IL-13), resulting in bone deposition [44,45].

Moreover, MKs produce a large amount of bone regulators (e.g., bone morphogenetic protein-2, -4, -6 [BMP-2, -4, -6], and TGF-β) [46] that provide for MK stimulation of bone formation. Platelet-derived growth factor (PDGF)-β and fibroblast growth factor (FGF), produced by MKs, promote OB differentiation and may help to maintain high bone mass [28].

MKs express receptors for various growth factors: TGF-β, PDGF, vascular endothelial growth factor (VEGF), and hepatocyte growth factor (HGF) [47,48,49,50]. They can be released from MKs and from stromal cells in the microenvironment. In this way, MKs can promptly respond to changes in the microenvironment and act on bone cells.

## 5. Role of MKs in Bone Metastasis

Debate surrounds the role MKs play in the development of bone metastasis. Studies have suggested a protective role of MKs against cancer metastasis [6,7,8]. Li and colleagues analyse the interaction between MKs and prostate cancer cells and demonstrate that the pre-treatment of human prostate cancer cells line (PC-3) with TPO prior to intra-cardiac inoculation in mice reduces bone metastasis formation. [6]. Since localization of mature MKs in the vascular sinusoids favours physical contact with the cancer cells during extravasation in the bone marrow, this might be important for the capacity of mature MKs to exert an inhibitory effect on metastasis colonisation. The interaction between MKs and prostate cancer cells in vitro is responsible for a decrease in the proliferation and an increase in apoptosis of carcinoma cells. However, this effect is specific for prostate cancer cells, as the interaction between MKs and the osteosarcoma cell line (SaOS2 cells) leads to the up-regulation of cancer cell growth [6]. 

Zaslavsky et al. report that thrombospondin-1 (TSP-1), a potent angiogenesis inhibitor, is up-regulated in the platelets of tumour-bearing mice consequent to the increase in mRNA for TSP in MKs and the increased number of MKs. TSP-1, produced and delivered by platelets, may play a critical role in suppressing tumour angiogenesis in the earliest stages of tumour growth. This represents a novel mechanism of host suppression of tumorigenesis (resulting from an increase in the number of circulating platelets and an increase in the megakaryocytic expression of TSP-1) [7]. 

In contrast, other observations argue against a protective role of MK in bone metastasis. It has been reported that MKs and the platelets priming the soil for the colonization of cancer cells in the bone marrow secrete an array of factors that influence haematopoietic niches. The release of a plethora of osteoclastogenic stimulators and inhibitors, OB promoters, mesenchymal growth factors, and bone matrix proteins is responsible for priming the soil. The release of pro-angiogenic factors (VEGF, PDGF, HGF, etc.) or of anti-angiogenic factors (TSP-1, plasminogen activator inhibitor, etc.) by MKs may act on the vascular niche [9,51].

**Figure 2:**
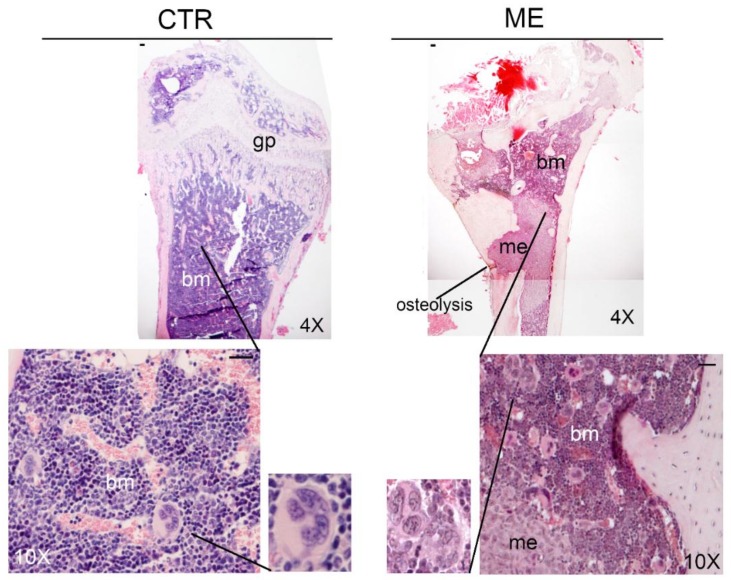
MK detection in bone marrow of control (CTR) and bone metastasis-bearing mice (ME). Representative images of haematoxylin and eosin (H&E) staining of femur sections in the context of our experiments, with the xenograft model of bone metastasis from breast cancer [52]. Three serial sections were analysed (*n* = 3). Statistical analysis is reported in Supplementary Materials (Table S1A). me: bone metastasis; bm: bone marrow; gp: growth plate; mk: megakaryocytes. Scale bar = 20 μm.

In the xenograft model of bone metastasis, obtained with intra-cardiac injection of breast cancer cells with high bone-tropism (1833 clone derived from MDA-MB231 cells) [52,53], we detected an increase in the number of MKs in the bone marrow of bone metastasis-bearing mice, as compared with control animals (13 ± 0.7 MKs/field and 5 ± 0.9 MKs/field, respectively at 26 days after intra-cardiac injection; Figure 2 and Supplementary Materials (Table S1A). Previous studies have reported similar results [8], and concluded that the increased number of MKs in bone marrow did not seem to be due to systemic factors released from the primary tumour, but rather as a response to the presence of cancer cells in the bone marrow [8]. Patients who died from metastatic breast cancer showed an increase in the number of MKs in the bone marrow, as found in the animal model [8].

The reservoirs of MKs are bone marrow, spleen, and to a lesser extent, lung, where the MKs are localized in the alveolar capillaries—areas with a high concentration of oxygen. Indeed, the number of MKs is higher in lung metastasis than in a normal lung [54]. The permanent siting of tumour emboli may stimulate the MKs to migrate to the lungs, where they augment the release of platelets into pulmonary circulation, events that facilitate the occurrence of metastasis. These observations shed further light on the role of MKs in metastasis, and indicate that their behaviour might depend on tumour type.

## 6. Megakaryocytes in the Bone Metastatic Environment of Xenografted Mice with Human Breast Cancer Cells

**Figure 3:**
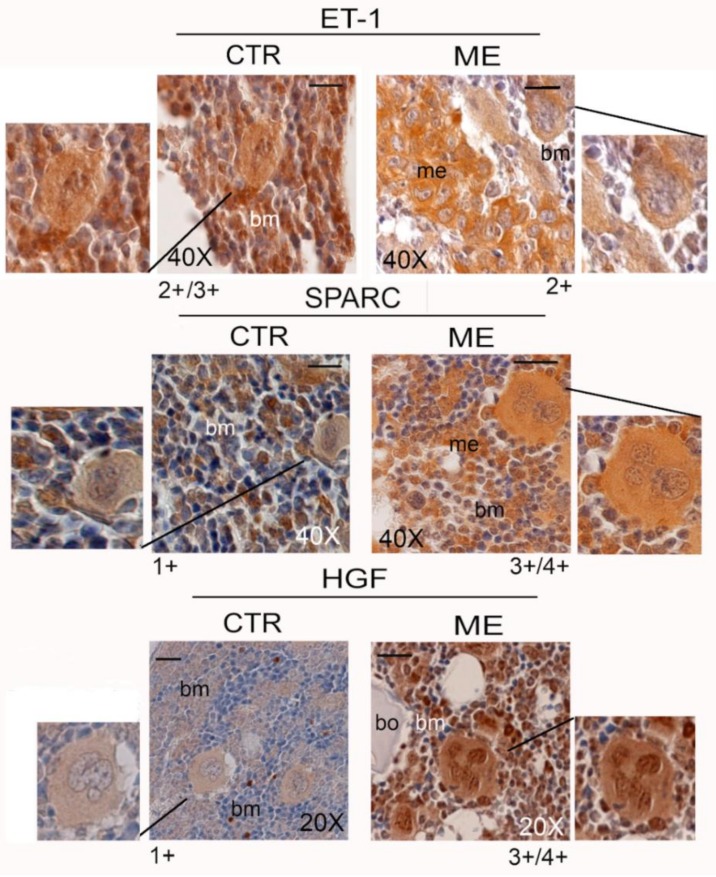
Positive reaction of MKs for endothelin-1 (ET-1), SPARC, and hepatocyte growth factor (HGF) in the bone marrow of femurs of control (CTR) and bone metastasis-bearing mice (ME). Representative images are shown, and three serial sections were analysed (*n* = 3). Semi-quantitative analysis of MK immunostaining is given below each panel: 4+ denotes very strong staining; 3+ strong staining; 2+ moderate staining; and 1+ weak staining. Statistical analysis is reported in supplementary materials (Table S1B). me: bone metastasis; bm: bone marrow; bo: bone. Scale bar = 20 μm.

In the context of our studies, in which we investigate the complex network of signalling in the bone metastasis microenvironment and the part that microenvironment plays in influencing metastatic growth, MKs provide an important source of biological stimuli, not only for the composition of platelet cargo, but also for the growth of metastatic cells. As shown in Figure 3, we demonstrate a positive immunoreaction of the MKs for endothelin-1 (ET-1), SPARC, and HGF in bone metastasis-bearing mice. 

The matricellular glycoprotein SPARC, derived from MKs and secreted into the ECM, might be necessary for colonization by metastatic cells and for osteoblastic niche formation. SPARC is able to mediate the interaction between carcinoma cells and ECM components, and to shape the epithelial–mesenchymal transition (EMT) [55]. ET-1 is a central player in the tumour microenvironment, where it exerts functions related to the migration and chemotaxis of neoplastic cells critical for invasiveness and dissemination. ET-1 is also implicated in conferring osteomimetic properties to metastatic cells. 

Also, HGF-scatter factor, a Met ligand, might participate in metastasis extravasation/engraftment, due to its role in the bone microenvironment at secondary growth sites [56]. In mice bearing bone metastasis, the expression of HGF was clearly evident in the MKs and the bone metastatic cells compared with the normal bone marrow, where the immunodetection of HGF was scarce within cellular components [50]. It is likely that not only MKs but also other mesenchymal and stem cells produced this biological stimulus required for the development of bone metastasis.

We hypothesized that the storage of SPARC, ET-1, and HGF in nascent platelets would modulate the “premalignant” platelet phenotype, with systemic effects on circulating tumour cells and favouring metastasis outgrowth [50]. Specifically, ET-1 and SPARC can have diagnostic significance for patients; both, in fact, are highly expressed in dysplastic lesions adjacent to breast carcinoma and in the corresponding bone metastases [57]. The HGF signalling pathway seems to be important for orchestrating bone colonization. Using a xenograft model of bone metastasis, we demonstrated that a four-kringle antagonist of HGF (NK4) reduced bone metastasis formation. Since the MKs release HGF, this might sustain their role in priming the soil [53].

Studying the role of MKs in human bone metastasis is difficult due to the scarcity of bone marrow obtained by biopsy. Nonetheless, we were able to detect the presence of MKs in small amounts of bone marrow in the biopsy. Analysis of bone marrow autopsy samples from patients who died from metastatic breast cancer demonstrated an increased number of polyploid MKs in a significant number of patients as compared with the controls [8]. The authors suggest that the increase in MKs was associated with a host protective response to metastatic cancer, while the loss of MKs permits the development of aggressive metastasis [8].

## 7. Conclusions and Perspectives

Overall, a complex regulatory interaction exists between MKs and bone cells. As concerns skeletal mass, MKs increase the bone mass through two mechanisms: secretion of factors that inhibit OC formation, and activity and stimulation of OB proliferation and differentiation.

Generalization of the role MKs play in bone metastasis as anti- or pro-metastatic is an oversimplification; the effect of a high number of MKs in metastatic tissue may differ with progression of the process. There remains much to learn about the interaction between MKs and bone cells. It would be useful to improve our knowledge on the behaviour of MKs in humans and to discover the context-dependent effects of MKs. We may speculate that during early stages of bone metastasis, MKs, due to their specific localization in close proximity to the vascular sinusoids, counteract the colonization of bone marrow by metastatic cells; this effect is related to their capacity to interact with cancer cells. In addition, the production of anti-angiogenic factors might control the growth of cancer cells. Subsequently, factors released from cancer cells (e.g., TNF-α, IL11, vascular cell adhesion molecule 1 (VCAM-1), matrix metalloproteinase-1 (MMP1), Jagged 1, and parathyroid hormone-related protein (PTHrP) [58]), by modifying the microenvironment, might affect the recruitment of MKs. In the new context, MKs may promote the development of bone metastasis because they are stimulated to secrete an array of cytokines that contribute to metastatic cell growth, thus preparing the “soil” (bone) and other factors that act on the “seed” (circulating tumour cells), rendering its phenotype appropriate for colonization.

## Supplementary Materials

The supplementary materials are available online http://www.mdpi.com/2073-4409/8/2/134/s1.
