# Supplementary material for: Megakaryocytes in Bone Metastasis: Protection or Progression?
Source: Cells. 2019 Feb 8;8(2):134. doi: 10.3390/cells8020134 (PMC6406759; doi:10.3390/cells8020134)
Supplement: Supplementary file 1 [file cells-08-00134-s001.pdf]

A

	CTR (n=3)	ME (n=3)
MKs/field	5 ± 0.90	13 ± 0.70**

B

Antibody	CTR (n=3)	ME (n=3)
ET-1	2.56 ± 0.18	2.00 ± 0.17
SPARC	1.10 ± 0.11	3.56 ± 0.18***
HGF	1.00 ± 0.17	3.56 ± 0.24***

A) Count of the of MKs in femur sections of control mice (CTR) and bone metastasis bearing mice (ME) after Haematoxylin & Eosin (H&E) staining of bone specimens. **B**) Semi-quantitative analysis of MKs in bone specimens of CTR and ME mice after immunostaining with the indicated antibodies. Both in A and in B, the statistical analysis (*t* test) was performed on three serial section (five fields for each section were randomly selected and counted under 200x magnification) for three mice for each treatment. The tables report the means ± S.E.; ** $p<0.05$; *** $p \leq 0.001$ *vs* respective CTR mice. Representative H&E staining and immunoistochemical images are shown in Figure 2 and 3.
